# LoRa-Based Traffic Flow Detection for Smart-Road

**DOI:** 10.3390/s21020338

**Published:** 2021-01-06

**Authors:** David Asiain, Diego Antolín

**Affiliations:** Escuela Universitaria Politécnica de la Almunia, C/Mayor 5, La Almunia de Doña Godina, 50100 Zaragoza, Spain; dantolin@unizar.es

**Keywords:** LoRa, LoRaWAN, Smart-Road, traffic flow, highway signaling

## Abstract

This paper presents a wireless traffic flow detection system, mainly focused on conditions in which the traffic flow is slow or stopped, which increases the risk of highway accidents. To achieve this goal, a Low Power Wide Area Network (LPWAN) based on LoRa called Short LoRa has been developed. This LoRa sub-network complies with the European Telecommunications Standards Institute (ETSI) harmonized standard for its compatibility in Europe countries. In addition, the development of the devices has allowed them to also work on a LoRaWAN network. The introduced development has been compared to a reference system mounted with laser barriers that provided a high accurate comparison. Field tests of the system have been carried out and the data obtained in the measurement has been analyzed with two different methods, and both of them were valid for the application. The results can determine vehicle speed with adequate precision at low speeds. The attenuating behavior of the communication signal is also analyzed through the Radio Signal Strength Indicator (RSSI). The relationship between vehicle speed, gate distances and RSSI attenuation has been studied. The system is proven to have efficient results in detecting traffic flow under the conditions for which it has been developed.

## 1. Introduction

In recent years, continuous advances have been made in radio frequency communications that allow low-power communications to cover long distances. This has caused the development of the Internet of Things (IoT), new low cost and energy efficient devices, using different new communication technologies. LoRa is a new promising (Low Power Wide Area Network) LPWAN, that permits communication in distances up to a few kilometers to not require the complex deployment and maintenance of multi-hop technologies [1]. LoRa-based networks have been set up and deployed in different applications from indoor [2] and urban [3] environments, to maritime [4] and mountain scenarios [5].

Nowadays, a growing research has focused on IoT based-applications, such as smart cities and smart roads. One application that is currently being researched currently is traffic flow detection.

In this last case, several sensor and detection techniques with specific advantages and disadvantages are used. A frequent approach for vehicle detection and classification is camera-based systems, which achieve a high classification success rate. This technique often requires a number of cameras to analyze the scenario from different angles and perspectives. In contrast, Hsieh et al. in [6] present an enhanced visual system which is able to arrange vehicles into distinct vehicle classes using a single camera. Regardless of the lower number of cameras, the use of these devices requires an additional effort in terms of installation, maintenance and privacy-related problems in real-world scenarios. Moreover, the success rate of these systems significantly decreases when the weather conditions impede the visibility. In order to improve the successful detection rate mixed camera systems have been developed, there are approaches using laser scanners [7], acoustic sensors [8], magnetometers [9] or accelerometers [10]. The first three approaches present similar drawbacks like camera-based detection systems; the last one requires construction works (pavement cut, etc).

Other techniques have proved to be accurate in detecting different elements in an environment, such as Radio Tomographic Imaging (RTI) [11], which uses a 2.4 GHz WiFi signal to locate people, similarly as it is proposed in [1] for an IoT application. The capacity of these systems grants an ability to monitor human motion by referring to the movement of their limbs [12]. Other works oriented to monitoring the traffic flow propose the use of different WiFi and ZigBee signals to detect traffic flow, classify the different vehicles, and even monitor their speed [10,13,14,15,16].

The reliability of LoRa is evaluated in [17] for different setup conditions. In outdoor experiments, it shows a correlation between temperatures, humidity, packet reception rate and the strength of the signal received. These environmental conditions have been considered in this project. In [18] a location method based on Radio Signal Strange Indicator (RSSI) using LoRa is evaluated.

There are smart road signs already set and running nowadays, and they will be seen frequently in a near future. The present work is focused on these type of signals, in which a communication of the signals and a synchronism between them is required, so that they might carry out the pertinent actions depending on the traffic situation. In this area, LoRa and LoRaWAN are appropriate communication technologies due to their relatively low consumption, low cost and long communication distances.

This present document displays an unexpensive flow or stopped traffic detection system in motorways based on LoRa. This is within the framework of a project to improve the signaling of a highway. The main project is focused on a signaling system for a dense fog situation that appears seasonally and that has the road section closed for long periods of the year. The system proposed in this article is an added functionality that—with zero material costs—allows the detection of stopped vehicles or vehicles with a reduced speed that produce a high risk of accidents with dense fog on the road.

The LoRa based network’s specific problem on the road it was developed for is the appearance of dense fog on the road, which causes a decrease on the drivers’ visibility, therefore being likely to spawn accidents. In these hazardous environmental conditions, other car detection technologies (cameras, radar, etc.) have shown difficulties in detecting the presence of dense and non-moving crowds of vehicles on the road obstructing traffic.

The paper is structured as follows: Section 2 provides a brief background to contextualize this work, the LoRa Network specifically proposed and the experimental deployment and cases of study. Section 3 comprises the experimental results and different data analysis methods. Section 4 includes the conclusions and future work.

## 2. Experimental Deployment (Materials and Methods)

This section describes the hardware and software development, explaining the different solutions tested: hardware and transceiver platform, network and data transmission setup and car detector algorithm.

### 2.1. Hardware Description

**Figure 1:**
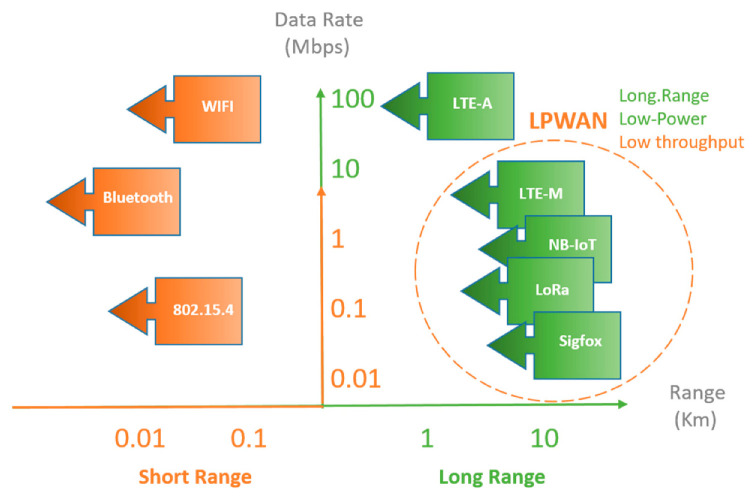
Comparison between Low Power Wide Area Network (LPWA) networks and other connectivity technologies.

The main objective of this job it is allowing to detect a slowed or stopped traffic on highways. In this way, the Radio Frequency (RF) transmission technology should have an extensive coverage, as well as being robust and low powered. Several RF technologies have been evaluated, such us, ZigBee, BLE (Bluetooth Low Energy), NB IoT, Sigfox, LoRa or LTE. Figure 1 shows the relationship between distance ranges vs. data transmission ratio.

On the other hand, the network topology is important to ensure a robust and simple communication protocol. The package integrity in this application it is very important for detect any stopped vehicle on the highway as soon as possible and give the corresponding advice. In addition, the road section be covered could have a few hundred of meters. To ensure the coverage, the transmission range should be greater than a kilometer. In this way, technologies as ZigBee and BLE are not appropriate since their transmission range is around a few hundred of meters.

In [19] the authors present a review of long-range technologies for IoT and the Table 1 shown a resume of the main attributes for each technology.

LTE is adequate for a high bandwidth and high data transmission ratio out of low power requirements. NB IoT, Sigfox and LoRa are LPWAN where these networks have a long-range data transmission also presents a low-power consumption. These protocols are suitable for the monitoring and signaling of highways.

NB-IoT can be deployed in different modes of operation. If deployed in guard band operation mode, it works in a frequency band similar to LTE, as indicated in Table 1. NB-IoT has a higher power consumption than LoRa.

Sigfox works well for simple devices and low data rate, as in this case. This protocol is not deployed everywhere, making it difficult to use. In addition, communication is better directed from the end point to the base station, and as it will be seen later in the work development, the application also requires communication between network nodes and the possibility of both uplink and downlink communication from the server or the gateway. Additionally, it has a small data rate and a short bandwidth.

LoRa allows you to configure and manage your own network, being a good option when bi-directionality is required because it has a symmetrical uplink and downlink connection. It also enables communication between nodes, although LoRaWAN does not contemplate it because LoRaWAN network topology is star. Lora allows an intermediate data transfer rate in comparison with the previous two. These characteristics make it appropriate for the application, so LoRa has been selected for the development of this work.

LoRa device architecture consists of the high-performance microcontroller, it is a low-power ARM^®^ Cortex^®^-M0 + based ATSAMD21G18 with 256 kB of flash, 32 KB of SRAM and operating frequency up to 48 MHz. It implements the LoRaWAN stack.

**Figure 2:**
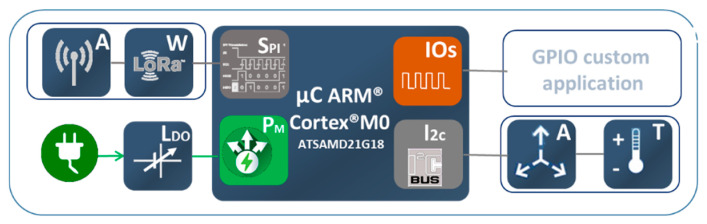
Shows the block diagram of the module, in the center the ARM M0 MCU, (**W**) LoRa transceiver, (**A**) omni-directional antenna, (**Ldo**) power supply and (**AT**) triaxial accelerometer.

The long range transceiver is the module RFM95 with a frequency of 868 MHz. Its main features are its high sensitivity below −148 dBm combined with the +20 dBm integrated power amplifier. Its modulation modes are FSK, GFSK, MSK, GMSK, LoRaTM and OOK. The connection interface between the microcontroller and the transceiver is SPI (Serial Peripheral Interface). Finally, the whole system is finished with an omnidirectional antenna of 1dBi gain. In addition, the module incorporates a triaxial MEMS lis3dh accelerometer and a general-purpose input-output connector. Lastly, the Power Supply consisting of a TPS7A05 low quiescent current low drop regulator. Figure 2 shows the block diagram of the module.

Up to this point, the most appropriate network protocol has been selected for the monitoring and/or control of the intelligent signaling of a highway. For traffic detection, it is necessary that the network has to be able to transmit at smaller distances to make a correct detection through the signal transmission power. This is why a short range wireless network is developed that is capable of coexisting with a LoRaWAN network, which we will call Short LoRa. This network will be presented in the next subsection.

### 2.2. Short LoRa Network Topology

**Figure 3:**
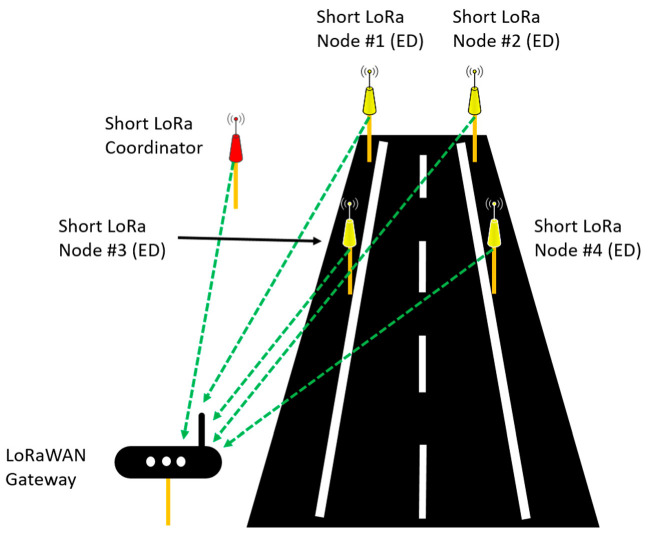
LoRaWAN standard topology.

Several network topologies have been studied in Wireless Sensor Networks (WSN). The most promising topologies consist of a mesh where all devices are peer to peer and there are no hierarchical relationships. This topology is very complex and presents high maintenance requirements. In this way, the star topology shown in Figure 3 is viable for this application due to the large transmission range, it is the topology used for LoRaWAN protocol. Star topology has less versatility communication options but it has small maintenance requirements, it is robust as it is not a multi-hop topology where the gateway receives the information from all End-Devices (ED) and control the network communications. This fact gives a high robust technology.

**Figure 4:**
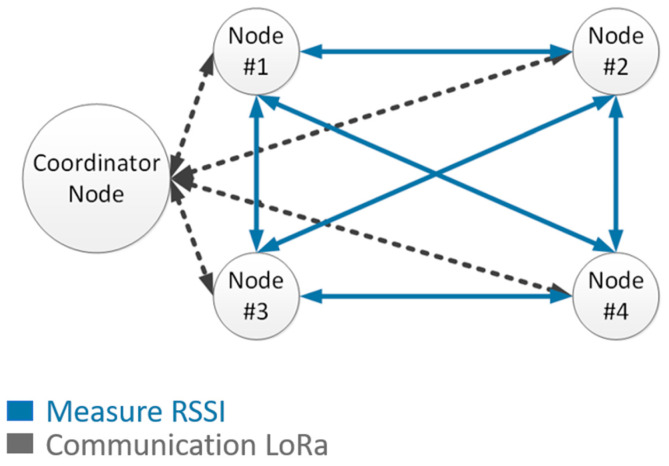
Short LoRa network topology.

The network proposed here works under a LoRaWAN network like the one presented in Figure 4 and it is implemented so that both networks can work together.

The Short Lora subnet requires five nodes, four of them are used to detect vehicle traffic and a fifth that does the work of network coordinator, mainly responsible for sequencing and establishing the work cycles for the measurements.

Following the LoRaWAN standard implementation Short LoRa use the same star topology, where the coordinator node is the frame sender in mode multicast, and this node is responsible for network synchronization and the final collection of data for further processing. The rest of the nodes (#1, #2, #3, #4) are promiscuous mode interface network for packet sniffing so that they can also measurement RSSI with each other and be able to detect vehicles. In addition, ED nodes are defined as Class A in LoRaWAN network, that according to LoRa [20] allows for bi-directional communications. Short LoRa sub-network use a specific single channel to work, which makes it different from the channels used by LoRaWAN. The specific operation of the network for vehicle detection will be presented in Section 2.4.

**Figure 5:**
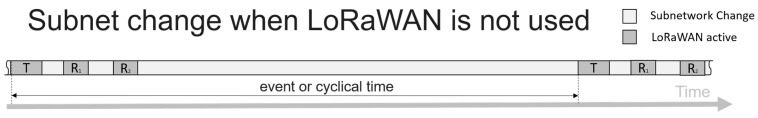
LoRaWAN vs. Short Lora transmission intervals.

The Figure 5 shows the LoRaWAN vs. Short Lora transmission intervals. It is observed that the idle periods of the LoRaWAN network are high, this fact is used to implement the Short LoRa network that will carry out the communications during these idle periods of LoRaWAN. To avoid data collisions, the subnet works on a different channel than LoRaWAN.

**Figure 6:**
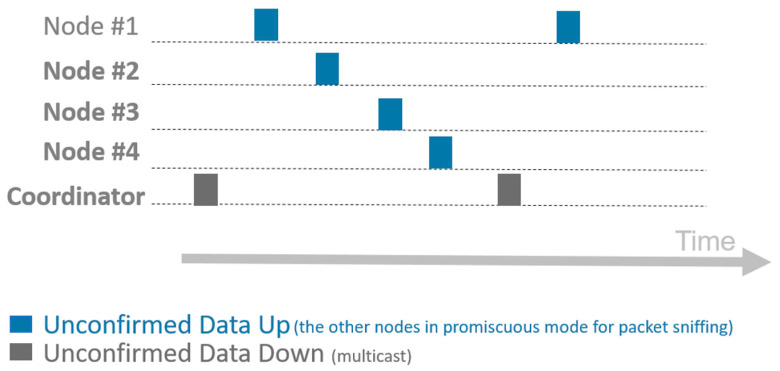
Short Lora transmission time diagram.

The transmission time diagram is shown in the Figure 6, in which we can see that the coordinating node of short Lora sends frame in multicast mode (a beacon signal) to synchronize the network and start the measurement, after which each of the nodes performs a multicast transmission to collect RSSI information from communication with the other nodes that are part of the subnet, sort LoRa.

### 2.3. Short Lora Network Protocol

The objective of the development of this application is the detection of stopped vehicles or slow traffic on Europe highways. The target region is important due to the restrictions presented by the regulations each one is subject to. In this case, it is regulated by EU Harmonized NRI for the 863–870 MHz band. The specific regulation for this kind of networks is given by the European Telecommunications Standards Institute (ETSI) ETSI EN 300 220-2 V3.2.1.33 [21].

On the other hand, it is necessary to establish a limit that we understand to determine a slow traffic speed. Assuming that 50 km/h (13.9 m/s) is slow traffic, and estimating the size of a vehicle of approximately 4 m. Based on these data, we can establish that the cycle time for the obstacle measurement with the radio frequency system exposed at the next point is 288 ms as seen in Equation (1).

(1) v = xt; t = xv = 4 m50 kmh = 4 m13.9 ms = 288 ms,

where: v is velocity, x is space and t is time.

Following the nodal distribution presented in [15] which has shown good results for other wireless communication technologies, but maintaining the LoRaWAN structure (for which 5 devices are required which will be justified later), it is necessary that each node transmit communicates every 50 ms.

On the other hand, the use of a standard LoRaWAN network has some work cycle and transmission power requirements that make it impossible to use this protocol in a standard way, as can be seen in Table 2.

Parameters given in Table 2, determine the minimum network transmission period. The theoretical analysis starts defining the time on the air, T_on-the-air_ (2), or packet transmission duration. This time determines in turn the minimum time that the network should remain without transmitting information.

(2) $$T_{\text{on-the-air}}\;=\;\left(n_{\mathrm{preamble}}{\;+\;n}_{\mathrm{payload}}\right)\cdot T_{\mathrm{symbol}}=\;\left(n_{\mathrm{symbols}}\right)\cdot T_{\mathrm{symbol}}$$

T_on-the-air_ depends on the number of symbols transmitted, including those of the preamble and the payload, whose sum is the number of symbols transmitted; and from the time necessary to transmit each of these symbols (T_symbol_). T_symbol_ is obtained from the rate of sending of symbols (R_Symbol_) which depends on the Spreading Factor (SF) and the signal bandwidth (BW).

(3) $$T_{\mathrm{symbol}}=\frac{1}{R_{\mathrm{symbol}}}$$

(4) $$R_{\mathrm{symbol}}=\frac{\mathrm{BW}}{2^{\mathrm{SF}}}$$

With these equations, the time on the air and transmission periods are shown in Table 3 for 6 bytes and the different network parameters.

As a result, the needs for traffic detection with a speed below 50 km/h, and considering the network limitations, in terms of the duty cycle and sampling period observed in Table 3, we can conclude that it is necessary to implement a subnetwork that can live with LoRaWAN. This subnetwork will work with 869.85 MHz, 125 kHz of modulation and SF7 5 mW (7 dBm) and a duty 100%, complying with the regulations indicated in ERC [21]. This network works out of LoRaWAN specifications but inside of ETSI EN 300 220-2 V3.2.133 regulation for Short Range Devices (SRD) [24].

In order to have disposable all channel time and not to affect the compatibility of the system with LoRaWAN, a subnetwork compatible has been implemented. To meet this requirement, the new network will base its implementation of the LoRaWAN protocol. Short LoRaWAN implementation vs. standard LoRaWAN are compared below.

**Figure 7:**
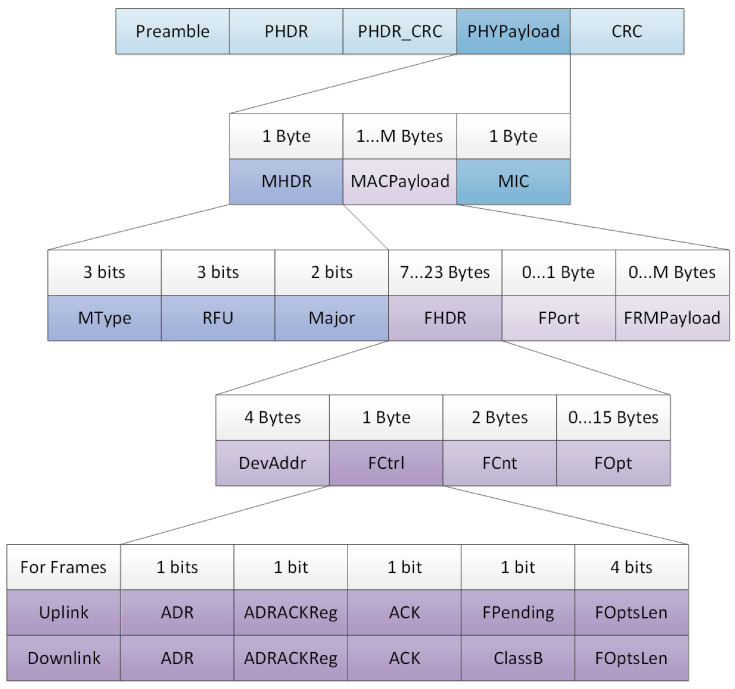
LoRaWAN packet structure [20,22].

Figure 7 shown the standard LoRaWAN Radio PHY layer message structure in order to establish a comparison line with the protocol proposed in this paper.

The LoRaWAN standard has a preamble value of 0 × 34, this parameter is used to establish a synchronized LoRaWAN network. The subnetwork proposed requires a different preamble, it is 0 × 12. The following parameters, PHDR and PHDR are used in explicit mode, default configuration for LoRa devices, but are transparent to the user and not used by them. For this reason, PHDR and PHDR_CRC have been simplified in Short LoRa, in the same way as MIC.

MHDR is made up of MType, RFU and Major. MHDR has been reduced only to a simplified MType, since the proposed network does not perform a negotiated network join or require an access code. The Table 4 shows differences between Standard LoRa MType and Short LoRa MType.

The next LoRaWAN parameter, FHDR, is composed by 4 bytes of device address, 1 byte of Frame Control, 2 bytes of Frame Counter and up to 15 bytes of frame options. In Short LoRa, these requirements have been reduced, FHDR is composed for 4 bits address (16 nodes), 2 bits for control (FCrtl) and 4 bits for count the payload length (FCnt). The 2 bits of control (FCtrl) are: the first one is the acknowledge (ACK) and the second is Reserved for Future Usage (RFU). In addition, Frame Options (FOpt) have been removed in order to simplify the network protocol.

The ADR and ADRACKReq fields within FCtrl are suppressed respect to the LoRaWAN standard because this subnet works by setting the data rate and transmission power.

The FOptsLen field within FCtrl and FOpts is suppressed because MAC commands can be sent in the FRMPayload field if the FPort field is set to 0.

Lastly, the payload frame (FRMPayload) contained the data collection that could be sent through the network, that could have up to 14 bytes. In this way using the encryption given by Advanced Encryption Standard (AES), we match the data packet sent to the minimum packet encoded by AES 128 bits. They must always be encrypted and must not exceed the maximum length of FRMPayload.

**Figure 8:**
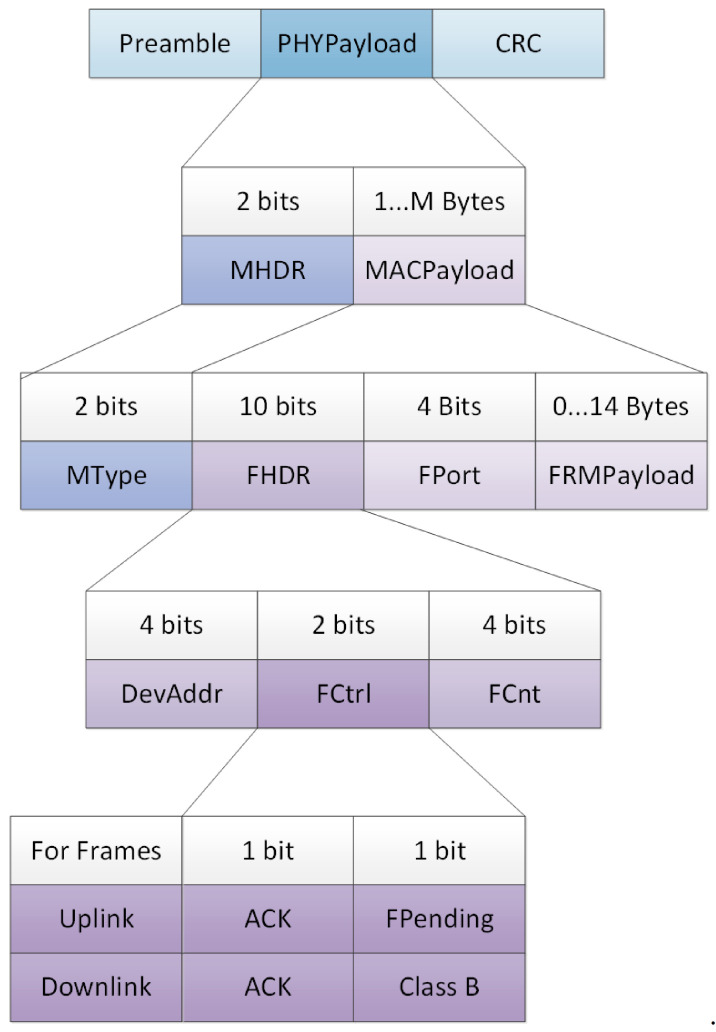
The proposed Short LoRaWAN packet structure.

The Figure 8 shows graphically the Radio PHY layer message structure proposed for Short LoRa.

The network nodes have been developed to work in Class A in order to they can be used for a standard LoRaWAN network when it is required by the application and the rest of the time you can work in the proposed low consumption network.

Under the proposed configuration, 869.85 MHz, 125 kHz of modulation and SF7, the different times in the air of the transmitted information have been calculated and it is shown in Table 5.

Although 50 km/h may seem like a low speed for detecting traffic on a highway, but this limit is justified below.

European ETSI regulation establishes a single channel to work on FSK within the LoRaWAN specification. It is true that working in FSK would allow the detection and measurement of higher circulation speeds corresponding to the Data Rate 7 (DR7) of the LoRa FSK specification with 50 kbit/s [22] compared to 11 kbit/s in LoRa SF7 (DR6) in the EU 863–870 MHz ISM Band. This limitation found for LoRa in speed measurement improves regulation in other countries such as the United States where using DR13 and SF7 the data rate is 21.9 kbit/s in US 902–928 MHz ISM Band.

Furthermore, analyzing the information presented in [25] where the LoRa communication is compared with the FSK communication, it can be deduced that as the bit rate increases, the LoRa sensitivity decreases, approaching the FSK sensitivity. Although, in this same document, we note that immunity against noise produced by other radio frequency signals is better in LoRa than in FSK. This is an important factor since it is intended to detect communication disturbances caused by obstacles in the direct line of sight and not by other RF signals.

On the other hand, in the datasheet of the RF transceiver used [26] explains the difference in the way of calculating the RSSI in both LoRa and FSK. In LoRa we have access to an average RSSI value of the sent packet while in FSK the RSSI value is smoothed on a user defined number of measured RSSI samples, the greater the number of samples, the greater the precision but the greater the delay in the measurement. This feature of LoRa is interesting since it allows us to work independently for each data packet, improving the robustness in the detection of obstacles.

Lastly, 50 km/h or lower speed on a high-speed traffic lane is considered a particularly risky situation. For this reason and given the results of the theoretical analysis carried out on the subnet protocol, where it is shown that the temporal conditions to detect vehicles at speeds of 50 km/h or less are given, and that therefore the proposed network is suitable for the application objective.

### 2.4. Short Lora Network for Traffic Flow Detection

The vehicle detection is based on the transmission losses or attenuation on communication transmissions. LoRa, and Short Lora send the Radio Signal Strength Indicator (RSSI) in each transmission. Therefore, it is assumed that the value of this signal will decrease when there is an obstacle in the direct transmission line or Line Of Sight (LOS).

Starting from the topology and the network protocol presented in Section 2.2 and Section 2.3, the operation of the vehicle detection system is developed, being the following:

1. The coordinator node sends a multicast synchronous service frame each 250 ms because the data transmission package is 6 bytes with a time on air of 36.10 ms, then a time slot of 50 ms is given to each node for its own communication avoiding collisions in this process. The system has a cycle frequency of 4 Hz. The beacon is the signal indicator to start the vehicle detection process.
2. When EDs receive the multicast synchronous service frame signal, the Node #1 time slot of 50 ms is opened. Node #1 sends a broadcast of 2 bytes and saves the RSSI given by the node coordinator, Node #2, Node #3 and Node #4. Afterwards, to save all the RSSI information, it is sent to the network coordinator in order to be processed when the detection cycle finished.
3. Next, Node #2 transmits in their time slot. First, it sends a multicast synchronous service frame, and after save the RSSI information and sends it to the coordinator node. This process is repeated for all End-Devices.

Finally, the information is collected by the LoRaWAN Gateway node, which works as the Short Lora coordinator in the interval that LoRaWAN is not transmitting. The collected information is processed on a personal computer. Processing techniques will be presented in the next section.

**Figure 9:**
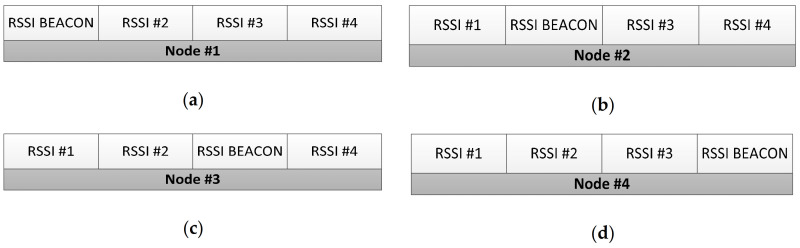
Radio Signal Strength Indicator (RSSI) transmitted for each End-Device to the network coordinator: (**a**) Node #1, (**b**) Node #2, (**c**) Node #3 and (**d**) Node #4.

The RSSI values transmitted by Node #1, Node #2, Node #3 and Node #4 are shown in Figure 9a–d, respectively.

### 2.5. Testbed

**Figure 10:**
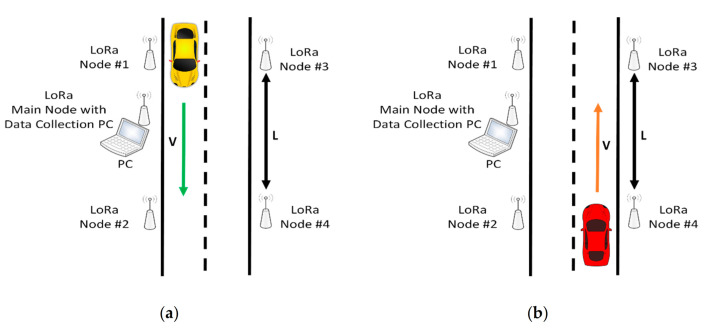

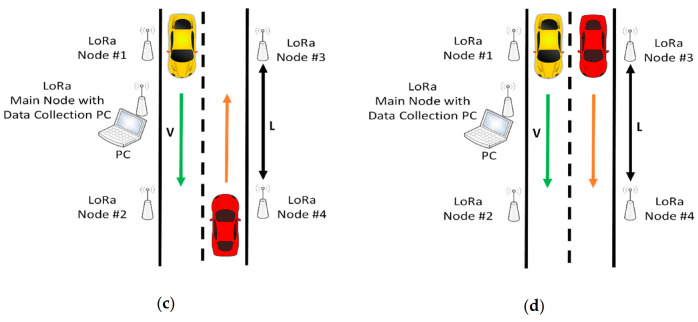
(**a**) One vehicle in one direction and its corresponding highway lane; (**b**) one vehicle in one direction and its corresponding highway lane in the opposite direction; (**c**) two cars in opposite directions; (**d**) two cars going the same way.

Different tests have been carried out in order to verify and validate the operation of the proposed system. The diagrams in the Figure 10 shows the configurations and the arrangement of the different elements of the vehicle circulation detection system on the road.

The tests represented in Figure 10a–d have been carried out in both directions of circulation since they are situations that can occur in roads in both directions.

All test have been carried out for several velocities: 10, 20, 30, 40 and 50 km/h and for two distances between the nodes that are in the same lane 10 and 20 m (Parameter V and L, respectively, in Figure 10).

In addition to these parameters and the tests carried out, it is necessary to explain the physical arrangement of the system and the nomenclature used in the description of the analysis. Both the communication and the spatial relationship between nodes #1–#2 (Gate 1) and #3–#4 (Gate 2), will be called gates, because these pairs of nodes are the “gates” through which the vehicle accesses the detection system. Similarly, the relations of both communication and spatial level of the connections of nodes #1–#4 (Crosses 1) and #2–#3 (Crosses 2) will be called “crosses” since they determine the X—shaped junction of the communication. Two more wireless connections are evaluated, nodes #1–#3 and #2–#4, these will be used later to determine miscellaneous communication losses, to determine the detection of the passage of vehicles in the controlled zone.

**Figure 11:**
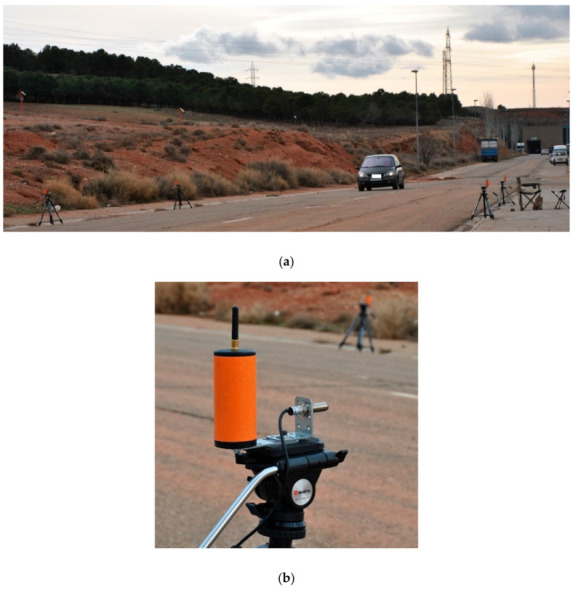
Pictures taken during the test: (**a**) Image taken during the test where the vehicle is going to enter the detection zone, in the measurement zone; (**b**) detail of Node#3 in a tripod during test.

The picture in Figure 11a shows the actual deployment made to carry out the implementation verification; while Figure 11b shows the detail of one of the deployed nodes. In it you can see that the electronics are inside a tube that is typically used in civil works on highways.

It should be noted that the communication links between the nodes that make up the gates include a laser-type optical path detector. This laser sensor allows us to compare the data that it provides us with the information extracted from power losses communication between nodes that form the pairs of each of the gates, to validate with greater precision the passage of a vehicle.

Another important point is the height at which sensor nodes have been placed. This should be above the height of the guardrail, typically 75 cm. To be able to adjust both the height to this requirement, and the alignment of the installed laser sensors to verify the passage of vehicles through the entrance gates to the LoRa detection system, topographic tripods have been used, as can be seen in Figure 6.

With this test setup, the data is acquired for subsequent analysis which is presented in the following section. To passes have been made for each of the speeds previously described in each of the configurations presented in Figure 10, at distances of 10 and 20 m between the gates, in order to have sufficient system information that allows checking viability of proposed system.

### 2.6. Power Consumption Analysis

**Figure 12:**
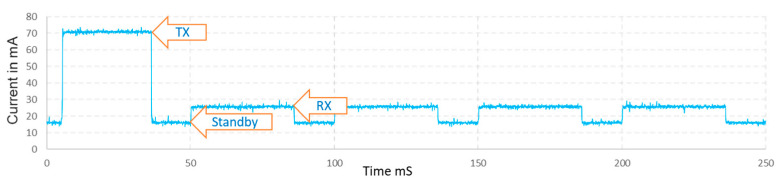
Current measured at 3.3 V in the complete cycle with an average current of 28.6 mA.

The measurement of the consumption of the network nodes shown in Figure 12, has been carried out with the devices working as described in Section 2.2 There are three different states. The Standby state, which we can define as the base current level with the MCU active and the LoRa modem in standby. The TX state, which corresponds to the consumption when transmitting whose difference value, is 53 mA at a power of 7 dB. Lastly, the Rx state that corresponds to the LoRa modem in receive mode with differential value is 10.2 mA.

The total average consumption of the entire cycle is 26.8 mA. It should be noted that this application works a duty of almost 100%. Furthermore, the transmission consumption is higher since the RFM95W module uses the RF output PA_BOOST and the consumption at this power is not optimized.

Generally, the consumption of a bright traffic sign with small LED diodes has a consumption of 5 W, while the application proposed here with 3.3 V of supply voltage and an approximate average consumption of 29 mA, give a lower power consumption at 0.1 W. This average consumption and the measured values presented in the graph of Figure 12 are within the ranges presented in [27]. We can conclude that the energy consumption that the proposed application adds to a luminous traffic sign does not significantly increase its consumption. Currently, the bottleneck in terms of consumption for luminous traffic signs is in the LED diodes.

## 3. Data Analysis and Results

Two methods of analysis have been proposed. The first of these is based on the detection of the derivative of signal loss, RSSI, when a vehicle passes. This method is relatively simple, but a priori it appears to be a fairly viable detection system.

The second method is more elaborate. It uses the loss of the RSSI value and the communication losses produced in what we will call free links, since there are not obstructions due to the movement of vehicles, to establish a base level on which to detect variations in transmission power. Free links correspond to communications between nodes #1–#2 and #3–#4.

The operation of both methods is detailed below in their corresponding subsections.

### 3.1. Derivative Data Analysis Method for Vehicule Detection

This method takes the RSSI data of the communications between the gates to the detection zone, Gate 1 formed by nodes #1–#2, and Gate 2 by nodes #3–#4. In addition, the differences in crosses will also be detected, that is, between the communications of nodes #1–#4 and #3–#2.

**Figure 13:**
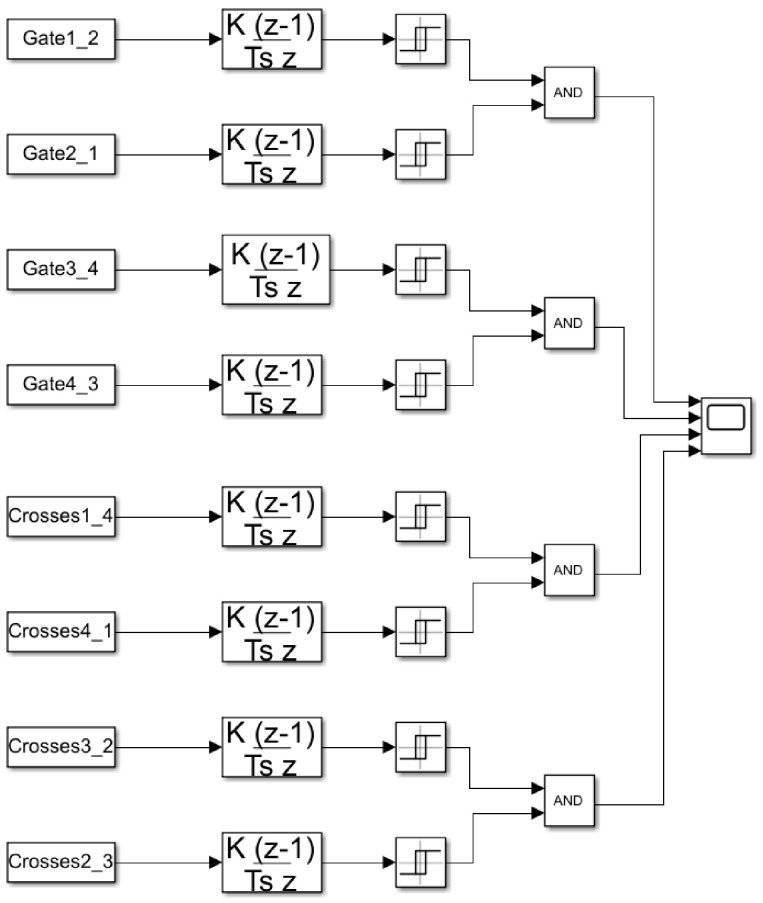
Simulink diagram for derivative data analysis method.

The acquisition of RSSI information is carried out as described in Section 2.3. As it is represented in Figure 3 and Figure 4, the communication and sending of the RSSI is bidirectional between each pair of nodes, this is shown in the Simulink analysis system diagram shown in Figure 13.

Using the transmission power measurement bi-directionally allows both RSSI data to be used, as seen in the figure above for each pair of nodes, making the possibility of detection more robust.

Figure 13 shows how the RSSI data of all bidirectional communications between pairs of nodes is processed and its discrete derivative is calculated. The value obtained from the derivative is entered in a comparator that when the derivative reaches a value of −5 dB, activates its output, giving it a ‘1′, otherwise the output is ‘0′ otherwise; Thus obtaining a digital encoding that with the ‘1′ indicates the presence of an obstacle in the transmission line and a ‘0′ when there is none.

The comparison value of –5 dB is established based on the collected experimental data, this being sufficient to detect the passage of vehicles without possible disturbances due to any noise or unwanted disturbance that modifies the RSSI value.

The results of the discrete derivatives of the pair-correlated transmissions of both the gates and the crosses in each direction are operated using the operation AND of Boolean logic, therefore, the passage of vehicles will only be detected if both results are ‘1′, that is, if the power losses in transmission are produced in both signals. This avoids errors in detection due to the power loss in only one of the signals produced by possible anomalous behaviors.

The results achieved are presented in Section 3.3, where they will also be compared with the other method of analysis proposed.

### 3.2. Link Budget Compensated Data Analysis Method for Vehicule Detection

This method is based on the detection of the difference of the communication signal of gates and crosses with a base level and reference level of bias. The method works as described below.

**Figure 14:**
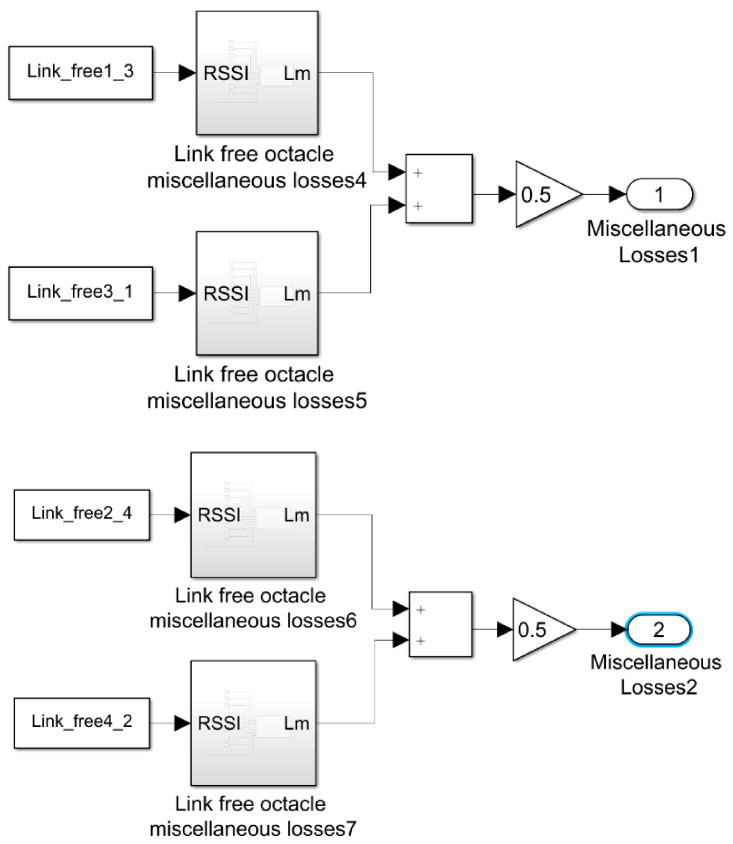
Miscellaneous losses calculation subsystem.

First, at the top of Figure 14, we can see the first two subsystems. These from the RSSI values and the theoretical losses obtained for communications between the free links or obstacle-free nodes #1–#3 and #2–#4, allow obtaining the existing losses due to other parameters that may intervene in communication and they are not controlled. This information will be used to compensate for these losses in communications at gates and crosses and to have an adequate base level or bias level.

For the theoretical calculation of communication losses, both the initial transmission power level and the elements that contribute gain to communication, such as antennas, and those that produce losses, such as cables and connectors, are taken into account. Free space losses have also been taken into account and are related to the distance that separates emitter and receiver as seen in Equation (5).

(5) $$L_{FS}=20\;log_{10}\left(4\pi \frac{d}{\lambda }\right)$$

where $L_{FS}\;$is path loss, usually free space loss (dB), λ is the signal wavelength and d is the distance between the antennas in the same units as wavelength.

This value of the power losses in the communication that we will call miscellaneous losses as seen in Equation (6), due to the possibility that they are produced by elements of unknown nature, is calculated in both directions of the same, that is, in the communication from node #1 to #3 and from #3 to #1, to later average it. The same is done for the communication of nodes #2 and #4.

(6) $$L_{M}=P_{Tx}+G_{TX}-L_{TX}-L_{FS}+G_{RX}-L_{RX}+P_{RX}$$

where: $L_{M}$ is miscellaneous losses (dB), $P_{Tx}$ is transmitter output power (dBm), $G_{TX}\;$is transmitter antenna gain (dBi), $L_{TX}$ is transmitter losses (dB), $L_{FS}$ is path loss, usually free space loss (dB), $G_{RX}$ is receiver antenna gain (dBi), $L_{RX}\;$is receiver losses (coax,connectors...) (dB) and $P_{RX}$ is received power RSSI (dBm).

**Figure 15:**
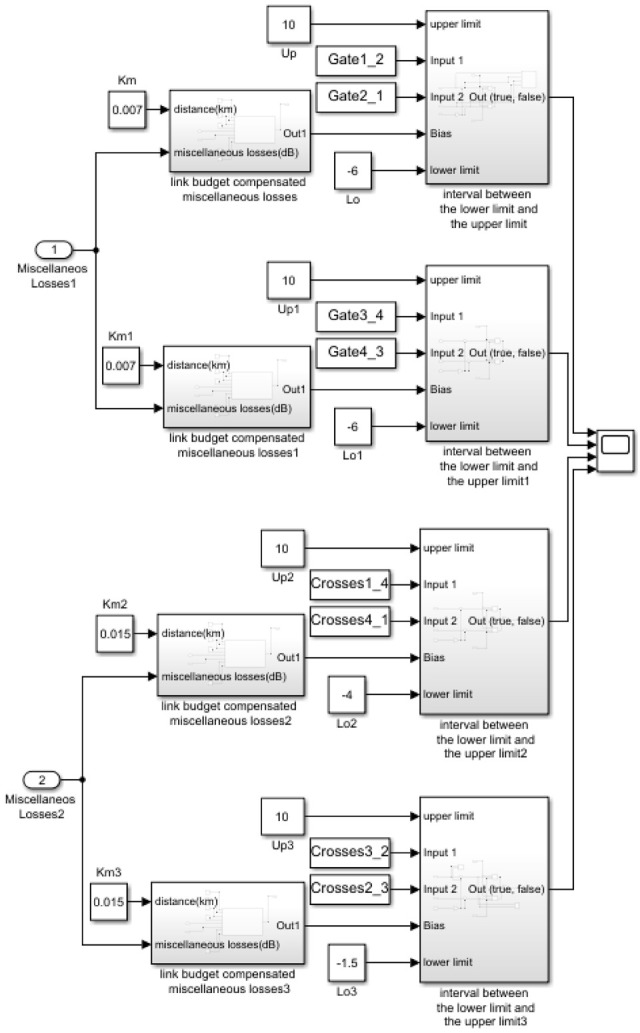
Simulink diagram for link budget compensated data analysis method.

Figure 15 shows the rest of the detection algorithm. In it we can see how the miscellaneous loss data calculated using the blocks in Figure 14 is entered into a new block. In this calculation block, the theoretical calculation of communication losses between gates or between crosses is obtained, taking into account the free space losses for the separation distance of these communications or these nodes. Miscellaneous losses are added to this calculation, which are assumed to be the same for all communications in the system, since they are within the same environment. With this, a base band or bias level is established on which to compare fluctuations in the reception power.

(7) $$P_{RX}=P_{Tx}+G_{TX}-L_{TX}-L_{FS}-L_{M}+G_{RX}-L_{RX}$$

where: $P_{RX}$ is received power RSSI (dBm), $P_{Tx}$ is transmitter output power (dBm), $G_{TX}\;$is transmitter antenna gain (dBi), $L_{TX}$ is transmitter losses (dB), $L_{FS}$ is path loss, usually free space loss (dB),$\;L_{M}$ is miscellaneous losses (dB), $G_{RX}$ is receiver antenna gain (dBi) and $L_{RX}\;$is receiver losses (coax, connectors...) (dB).

Finally, this bias reception power ($P_{RX}$) is subtracted from the RSSI values received at each of the bidirectional gates and crosses reception values, as appropriate. With this compensated base level the new base level is around zero, two limits are set, one upper and one lower. The chosen limit values have been obtained experimentally for each communication pair. When one of the signals crosses the lower limit it produces a logical ‘1′. In this method, unlike the previous one, the output value of the comparison is passed through an OR logic gate so that when one of the two RSSI signals produces a positive, it is detected. If this detection exceeds the maximum time established based on the length of the vehicle and the minimum speed, we face slow or stopped traffic.

This detection is carried out at each of the gates and crosses. The results will be explained in the next subsection.

### 3.3. Results

**Figure 16:**
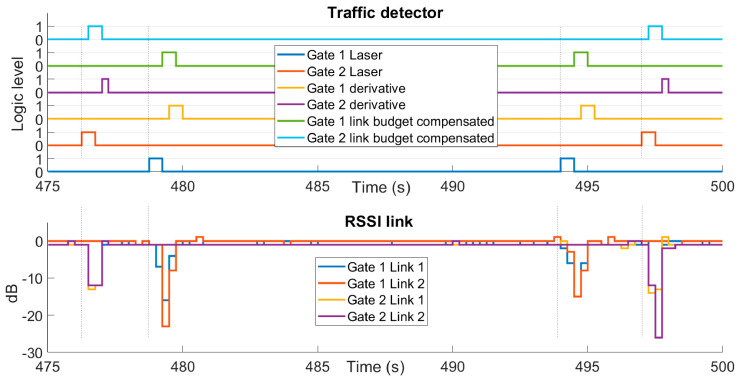
Above, it is shown the digital signals achieved after to apply the data analysis method compared with laser signals. Below are RSSI signal variations over 0 dB. Both graphics correspond with 30 km/h of car velocity and 20 m between gates.

Figure 16 shows the results of the vehicle detection algorithms. For the comparison and analysis of the results, the data obtained by means of the two laser barriers located between the links of the gates are taken as a reference. The response time of these laser barriers has a high accuracy, providing an adequate reference for system validation.

The case presented in Figure 16 corresponds to one of the tests carried out in which the vehicle runs at 30 km/h and the distance between gates is 20 m. In the test, the car enters the detection zone twice, the first of which enters through the gate that we will now call Gate 2, formed by nodes #3 and #4, and exits through what we will call Gate 1, corresponding to nodes #1 and #2.

In the figure we can see several things, the first of which are laser barriers that provide an answer first, consistent with its use as a reference system. Next, we observe that the link budget compensated method has a faster and equal detection on both gates; while the method based on the discrete derivative responds somewhat later and with signals of non-uniform width.

Despite the differences shown, the two data processing methods are equally valid for detecting the passage of vehicles.

The lower graph of Figure 16 shows the attenuations produced in the RSSI value with the passage of vehicles with respect to an attenuation level of 0dBs. The attenuations of the transmissions of both gates and of the bidirectional communication of the pairs of nodes that form each of them are presented separately. The graph refers to these signals as Gate 1 Link 1, this being the communication from node #1 to #2; Gate 1 Link 2 from #2 to #1; Gate 2 Link 1 from #3 to #4 and Gate 2 Link 2 from #4 to #3, thus representing all the detection signals present between the gates communications.

The RSSI data for this analysis is collected in Table 6, both at gate and crosslinks. Studying what this attenuation looks like shows that the gate signs are more attenuated than the crossing signals. This reduction in attenuation is due to the fact that in the crossings there are more deflations, more rebounds and; therefore, less attenuation and less sensitivity.

On the other hand, the average gate attenuation is at least 15 dB, and this RSSI attenuation value occurs in both directions of communication. These two characteristics make the system very robust.

Crossings can be used to sense the direction of the vehicle or to make the gate detection algorithm more robust. Although the detection system works satisfactorily using only the gate signals.

For unobstructed links (#1–#3 and #2–#4), they have a signal RMS attenuation of 10 dBs and RMS noise of 1 dB.

Based on the RSSI information processed and the corresponding digital signal obtained for each data analysis method, together with the reference laser system, the time it takes for a vehicle to pass from one gate to the next has been measured. From the information shown in Figure 16 the pulse instants are obtained for the calculation of the time difference. Since the distance between the gates is known, which is fixed for each case study (10 and 20 m), and the time it takes for the vehicle to travel that gap is measured, the speed of the car can be calculated.

Table 7 shows these results. In the table, when the vehicle enters through Gate 1 and exits through Gate 2, it is considered positive. The opposite direction is considered negative (Gate 2 to Gate 1).

From the information presented, both for 10 and 20 m of gate distances, it is observed that for low speeds of 10 to 30 km/h the measurement through the wireless network is quite accurate and its response time is quite fast compared to the reference measurement obtained using the laser barriers. The accuracy decreases as the speed increases and this is due to the sampling time with which the network works. This aspect can be improved, but it would be necessary to enter an operating mode outside the ETSI EN 300 220-2 standard. The other option is to work in FSK mode that conflicts with the working modes of LoRaWAN gateways, compromising compatibility between LoRaWAN and Short LoRa.

With the data shown in Table 7, the absolute and relative errors in the measurement were calculated for both methods, always taking as a reference the measurement obtained from the laser barriers. The error values are shown in Table 8.

In general, it can be seen that the error increases with speed and is greater when the distance between gates is 10 m, since the time it takes for the vehicle to cross both tares is less than in the case of 20 m. As already mentioned, this is produced by the sampling time. It can also be seen that the error in the derivative method is generally less than in the link budget compensated method.

### 3.4. Dicussion

We can find in the literature different communication protocols for the Intelligent Transport System (ITS). Two of these protocols, perhaps the most relevant in this area are: 6LoWPAN and IEEE 802.11p. In [28,29] the authors talk about the use of the 6LoWPAN and IEEE 802.11p protocols, respectively, in ITS applications.

Both protocols have greater potential for these applications, as they allow Vehicle-to-Vehicle (V2V), Vehicle-to-Infrastructure (V2I) communication, as well as global communication via the Internet of Things (IoT). On the other hand, these protocols require a greater infrastructure and that the vehicles have a compatible communication system.

The implementation of this type of system is much more complex and at present in the target country of the project, it is not feasible to develop a communication network of this type, involving both the road communication/monitoring infrastructure and the vehicles that circulate through it.

The objective of the proposed work is much more modest, in this sense, and aims to monitor the condition and climate of a highway, leaving aside the information from the vehicles. The main objective is to be able to detect an accident or a situation of collapse on a motorway, with extreme importance in circumstances in which weather conditions can hinder visibility during traffic on the road, increasing the risk of an accident. This is without the need for vehicles to carry a communication system compatible with that installed on the road, that is, it is a system solely intended to monitor the condition and environment of the road that uses the communication signal itself to detect circumstances in which may be a vehicle stopped or moving slowly on the road.

This work presents a traffic detection system based on a LoRa network that works with a communication frequency of 868 MHz. We can find in the literature other works of a similar nature that use WiFi communication technologies, IEEE 802.11b [14,15], IEEE 802.11n [13] or Low Power Wireless Sensor Networks (LPWN) as IEEE 802.15.4 [16] with communication frequencies in the 2.4 GHz band. Both communication frequencies are within the Industrial, Scientific and Medical (ISM) bands.

These works are oriented for application in cities, places where 2.4 GHz wireless networks are abundant and whose infrastructure can be exploited. The results presented by the cited authors are promising. The problem with this frequency is its short range, such networks are no longer available on a highway, and due to their short range they present certain problems.

In this work, the design of a network system based on LoRa with a communication frequency of 868 MHz is proposed, this allows communication over long distances, although in the work a short-range subnet based on LoRa is proposed, its capacity coexisting and working with LoRaWAN means that these small subnets can be distributed and controlled by a single LoRa Gateway.

Regarding the algorithms in the treatment of the data, they use Support Vector Machines (SVM) [14,15,16] and machine learning algorithms such as k-Nearest Neighbors (k-NN) [13]. These algorithms for data analysis allow them to classify the type of vehicle is going. The algorithms proposed in the article are simpler but just as effective in detecting the passage of vehicles.

Table 9 shown a comparative summary of different communication protocols discussed in this subsection.

With the information set out in this subsection, we can conclude that the proposed system, with simple calculation algorithms, has satisfactory results for the detection of vehicle traffic, although with certain restrictions when it complies with the European regulation ETSI EN 300 220-2 V3.2.133. These restrictions mainly affect when calculating the speed estimate at speeds above 50 km/h, since it is at the limit of what the subnet sampling time allows.

## 4. Conclusions

A new LoRa Network Protocol has been developed. It is presented in Section 2. This protocol is compatible with LoRaWAN and can live and work with this infrastructure at the same time on the devices. The design allows the device to work on both networks with the same transceiver.

The network protocol proposed in this paper works is a Personal Area Network (PAN), so the power consumption for work in this mode is lower than for a LoRaWAN; a LoRa (Long Range) network is a Low-Power Wide-Area Network (LPWAN) technology.

The network has been deployed in a road and tests have been carried out to validate the traffic flow detection system based on Radio Signal Strange Indicator (RSSI) in LoRa Networks. The test validates different cases: different vehicle speeds, one vehicle on the road, two vehicles in the same way and two vehicles in opposite directions.

A power consumption analysis has been carried out in Section 2.5. It shows how the consumption of the application proposed in this work does not present a significant increase in a luminous traffic signal.

Two data processing systems have been tested. The first one is shown in Section 3.1. It is based on the RSSI derivative value of the communication signal compared to a threshold value. It is a simple but effective method, although it has the limitation of not being able to detect stopped traffic.

The second method is more complex, in this case, it is possible to detect stopped traffic. For its operation, the communication losses are calculated based on the RSSI data between the nodes whose communication is not interfered with by the flow of traffic, nodes #1 and #2 and nodes #3 and #4.

The system is synchronous, making it possible to obtain the speed of the vehicles that are traveling through the monitoring area. This allows the development of traffic signs that adapt their response to the speed of vehicles on a road.

Field tests have been carried out that have allowed an in-depth analysis of the system: calculating the speed of the vehicles in the tests, as well as the errors in this calculation thanks to having laser barriers as reference; and the RSSI attenuation has been analyzed for the different communication links.

In all test cases for both analysis methods, the results shown in Section 3.3 are successful.

LoRa is a promising technology in IoT, used to develop a new WSN for the improvement of highway safety. A slow and stopped traffic detection system has been developed based on the measurement of intensity of RF signals, using RSSI. This allows these hazardous road conditions to be detected when visibility conditions are reduced, such as dense fog.

It is deduced from the results obtained that the system proposed in this work is adequate for the detection of slow or dense vehicle traffic.

In the future, the information of the intersections (Crosses) can be processed to obtain the direction of movement of the vehicles.

## Figures and Tables

**Table 1:** Attributes for long-range technologies for the Internet of Things (IoT).

Attribute	LTE-M	NB-IoT	Sigfox	LoRa
Frequency Band	700–900 MHz	700–900 MHz	868, 902 MHz	Sub-GHz ISM
Data Rate	375 kbps	25–65 kbps	0.1 kbps	0.3–37.5 kbps
Bandwidth	1.08 MHz	200 kHz	100 Hz	<500 kHz
Range	<15 km	<35 km	Rural: 30–50 km Urban: 3‒10 km	Rural: 10–15 km Urban: 3‒5 km

**Table 2:** Bands and regulations according to the European Reseach Council (ERC) Recommendation 70-03 and LoRaWAN Specifications [20,21,22,23].

Band Number	Frequency (MHz)	Duty Cycle	Power
g0	865.0–868.0	1% or LBT + AFA ^1^	25 mW = 14 dBm
g1	868.0–868.6	1% or LBT + AFA	25 mW = 14 dBm
g2	868.7–869.2	0.1% or LBT + AFA	25 mW = 14 dBm
g3	869.4–869.65	10% or LBT + AFA	500 mW = 27 dBm
g4	869.7–870.0	1% or LBT + AFA	25 mW (no duty-cycle requirement if power < 5 mW/7 dBm)

^1^ LBT + AFA: Listen Before Talk (LBT) with Adaptive Frequency Agility (AFA).

**Table 3:** Network configuration vs. duty cycle, time on air and data transmission period with 6 bytes of data transmission.

Frequency [MHz]	Power [mW]	Modulation [kHz]	Spreading Factor	Time on Air [ms]	Duty Cycle [%]	Cycle Scan Network [s]
868.3	25	250	SF7	18.05	1	1.805
869.525	25	125	SF9	123.90	10	1.239
869.525	500	125	SF7	36.1	1	3.61
868.8	25	125	FSK	3.871	1	0.386
869.850	5	125	SF7	36.1	100 ^1^	0.1805

^1^ With a spectrum access technique such as LBT or equivalent and a maximum transmit period of 1 min for each transmission.

**Table 4:** Media Access Control (MAC) message types.

Standard LoRa MType Value	Short LoRa MType Value	Description
000	Not Available	Join Request
001	Not Available	Join Accept
010	00	Unconfirmed Data Up
011	01	Unconfirmed Data Down
100	10	Confirmed Data Up
101	11	Confirmed Data Down
110	Not Available	RFU
111	Not Available	Proprietary

**Table 5:** Time On Air for 869.850 MHz, modulation frequency 125 kHz and spreading factor SF7.

Number of Bytes	Time on Air [ms]
2	30.98
6	36.10
16	51.46

**Table 6:** RSSI measurements from communication transmissions during the test with 20 m of distance between gates.

Connection	Max. Signal (dB)	Min. Signal (dB)	Mean Signal (dB)	RMS Signal (dB)	RMS Noise (dB)	RMS SNR ^1^
Gates Distance 20 m						
Gate1(#1–#2)	27.00	8.00	16.54	16.96	0.46	16.50
Gate1(#2–#1)	27.00	7.00	15.46	16.13	0.51	15.62
Gate1(#3–#4)	27.00	10.00	16.21	16.65	0.94	15.71
Gate1(#4–#3)	26.00	8.00	15.95	16.50	1.00	15.50
Mean Gates	26.75	8.25	16.04	16.56	0.73	15.83
Crosses1(#1–#4)	19.00	6.00	8.83	9.44	0.35	9.09
Crosses1(#4–#1)	16.00	6.00	8.96	9.42	0.85	8.57
Crosses2(#2–#3)	12.00	6.00	7.14	7.34	0.73	6.60
Crosses2(#3–#4)	20.00	6.00	7.63	8.30	1.21	7.09
Mean Crosses	16.75	6.00	8.14	8.62	0.79	7.84
**Gates Distance 10 m**						
Gate1(#1–#2)	29.00	14.00	19.68	19.89	0.20	19.69
Gate1(#2–#1)	29.00	13.00	19.71	20.11	0.50	19.61
Gate1(#3–#4)	33.00	9.00	18.00	19.01	0.97	18.03
Gate1(#4–#3)	28.00	10.00	16.95	17.61	0.53	17.08
Mean Gates	29.75	11.50	18.58	19.15	0.55	18.60
Crosses1(#1–#4)	22.00	7.00	12.34	13.01	0.40	12.61
Crosses1(#4–#1)	24.00	8.00	12.03	12.62	0.31	12.31
Crosses2(#2–#3)	24.00	7.00	11.64	12.63	0.42	12.21
Crosses2(#3–#4)	19.00	6.00	9.92	10.43	0.63	9.80
Mean Crosses	22.25	7.00	11.48	12.17	0.44	11.73

^1^ SNR: Signal Noise Ratio.

**Table 7:** Data analysis and speed estimation for the different processing methods.

	Laser Measurements		ShortLoRa Network Measurements (Derivative Method)		ShortLoRa Network Measurements (Link Budget Compensated Method)	
Car Velocity (Km/h)	Differential Gates Time (s)	Velocity (km/h)	Differential Gates Time (s)	Velocity (km/h)	Differential Gates Time (s)	Velocity (km/h)
Gates Distance 20 m						
10	6.77	10.63	6.8	10.58	6.8	10.58
10	−6.78	−10.61	−6.5	−11.07	−6.7	−10.74
20	3.54	20.32	3.8	18.94	3.5	20.57
20	−3.78	−19.04	−3.7	−19.45	−3.7	−19.45
30	2.59	27.71	2.8	25.71	2.5	28.8
30	−2.54	−28.27	−2.5	−28.8	−2.2	−32.72
40	1.92	37.42	2	36	2	36
40	−1.87	−38.35	−1.7	−42.35	−1.8	−40
50	1.51	47.58	1.5	48	1.5	48
50	−1.51	−47.65	−1.5	−48	−1.3	−55.38
**Gates Distance 10 m**						
10	3.69	9.92	3.5	10.28	3.7	9.72
10	−2.92	−12.32	−2.8	−12.85	−2.7	−13.33
20	1.92	18.73	2	18	2	18
20	−1.93	−18.65	−1.7	−21.17	−1.7	−21.17
30	1.27	28.27	1.3	27.69	1.5	24
30	−1.21	−29.72	−1.3	−27.69	−1	−36
40	0.93	38.37	1	36	1	36
40	−0.93	−38.66	−0.7	−51.42	−0.8	−45
50	0.72	50.06	0.8	45	0.7	51.42
50	−0.75	−48	−0.8	−45	−0.6	−60

**Table 8:** Average absolute and relative errors for both car detection methods.

	Derivative Method		Link Budget Compensated Method	
Car Velocity (Km/h)	Absolute Error	Relative Error	Absolute Error	Relative Error
Gates Distance 20 m				
10	0.05	0.004	0.05	0.004
10	0.46	0.04	0.13	0.01
20	1.38	0.07	0.24	0.01
20	0.42	0.02	0.42	0.02
30	2.00	0.07	1.09	0.04
30	0.52	0.02	4.45	0.16
40	1.42	0.04	1.42	0.04
40	3.99	0.10	1.64	0.04
50	0.41	0.01	0.41	0.01
50	0.35	0.01	7.73	0.16
**Gates Distance 10 m**				
10	0.37	0.04	0.05	0.02
10	0.53	0.04	0.46	0.08
20	0.73	0.04	1.38	0.04
20	2.52	0.14	0.42	0.14
30	0.59	0.02	2.00	0.15
30	2.04	0.07	0.52	0.21
40	2.38	0.06	1.42	0.06
40	12.76	0.33	3.99	0.16
50	5.07	0.10	0.41	0.03
50	3.00	0.06	0.35	0.25

**Table 9:** Comparative summary of different communication standards.

Classification	Low Power Wireless Personal Area Network (LWPAN)	Wireless Access Spaces for Vehicular Environment (WAVE/DSRC)	Low Power Wide Area Network (LPWAN)
**Standard**	IEEE 802.15.4	IEEE 802.11p	LoRa
**OS**	No	Yes	No
**Range**	10–100 m	100–1000 m	1000–10,000 m
**Power**	Low	Medium	Low
**High Layers**	ZigBee, 6LoWPAN	IPv6, WSMP (WAVE Short Message Protocol)	LoraWAN
**Modulation Type**	BPSK, OQPKS	BPSK, QPSK, 16QAM, 64QAM	LoRa, FSK
**Bit Rate (Mbps)**	0.020 to 0.25	3 to 27	0.003 to 0.050
**Frequency Bands of Operation**	868 MHz, 915 MHz and 2.4 GHz	5.9 GHz	433 MHz, 868 MHz and 915 MHz
**Network Architecture**	Peer-to-peer or star networks	Peer-to-peer ad hoc network in topology and location based	Star-of-stars topology in which gateways
